# Commentary on: Microautologous Fat Transplantation for Primary Augmentation Rhinoplasty: Long-Term Monitoring of 198 Asian Patients

**DOI:** 10.1093/asj/sjw059

**Published:** 2016-04-19

**Authors:** Woffles Wu

**Affiliations:** Dr Wu is a plastic surgeon in private practice in Singapore

Injection rhinoplasty using a variety of fillers delivered through fine needles or cannulae is currently one of the most popular procedures for nasal augmentation throughout Asia.^1-4^ It is a convenient procedure that takes under 10 minutes to achieve, requires no operation or anesthesia, has minimal downtime, and is immediately satisfying with predictable results. It is the current gold standard against which other injectable techniques for nasal augmentation are measured. The fillers can also be used to create a continuous and smoothly curved naso-orbital line that enhances nasal and periorbital aesthetics, something that a surgically placed silicone implant cannot achieve.

The authors are to be commended for a very well written paper describing in detail the use of a novel device, the microautologous fat transplantation (MAFT) gun (MAFT-Gun, Dermato Plastica Beauty Co, Ltd, Kaohsiung, Taiwan), to deliver via injection, small parcels of autologous fat to the dorsum of the nose in 198 patients requesting nasal augmentation.^5^ The technique and rationale for using it is well described and it would be easy for any physician to understand how to use this device. Such patients are always receptive to exploring minimally invasive techniques since traditional surgical rhinoplasty using synthetic materials such as silicone have their fair share of unwanted side effects. Whilst strictly still an operative procedure, the MAFT gun technique is certainly minimally invasive and is a useful alternative option if the patient wishes to avoid a more complex surgical procedure.

Numerous injection guns for autologous fat transplantation have been used in the past but the MAFT gun appears to be more refined as it has been designed and calibrated to deliver parcels of fat as small as 0.0056 mL to 0.0067 mL under low pressure. This may be an important consideration in reducing periorbital vascular complications and the devastating consequence of visual loss.

The method of fat preparation employed by the authors is that of Coleman^6^ with centrifugation of the harvested fat at 3000 rpm for 3 minutes with no additional maneuvers to emulsify or pre mash the fat into smaller particles. The authors deliver their injections through a blunt 18G cannula that has to be inserted via a stab incision at the tip of the nose which is subsequently stitched. This may have the potential to create a hyperpigmented scar in certain individuals. It was not mentioned why an 18G cannula was chosen instead of a smaller cannula since the parcels of fat delivered are so small and whether the authors encounter frequent blockages during the delivery of the fat injections.

The technique appears sound and logical but the results shown are extremely subtle with only a vague improvement in dorsal height or shape and it is wondered whether this is due to patient preference or a limitation of the technique.

The paper^5^ is laid out systematically but there is one significant omission which needs to be addressed: a discussion on the relevant anatomy of the nose, in particular its vasculature and how to avoid a serious complication of vascular occlusion and visual loss.

Beleznay et al^7^ reviewed 98 cases of documented or published blindness from the world literature of which approximately half of these cases were due to the injection of autologous fat whilst the other half were due to the injection of filler materials. Overwhelmingly however in both categories, blunt cannulae were used for injections into the nose, glabellar, and forehead regions (76.5%). Ninety-eight people with blindness as a result of a cosmetic procedure is a most alarming statistic. It calls into question the use of cannulae in this region and whether a sharp needle technique is safer.

Wu^8^ has previously described the Asian nasal anatomy and its vasculature. There are 5 different tissue planes overlying the osseocartilaginous framework of the nose (namely from superficial to deep): (1) the skin; (2) the superficial or subcutaneous areolar layer; (3) the fibromuscular layer; (4) the deep areola layer; and (5) the periosteum or perichondrium depending on where the tissue has been sampled. The major blood vessels of the nose lie on the fibromuscular layer as paired branches (the alar and columellar arteries) and interconnecting plexuses derived from the facial artery (external carotid system) as well as the dorsal nasal artery (internal carotid system). A watershed exists in the midline all the way down to the tip and columella (Figure 1).

**Figure 1.**
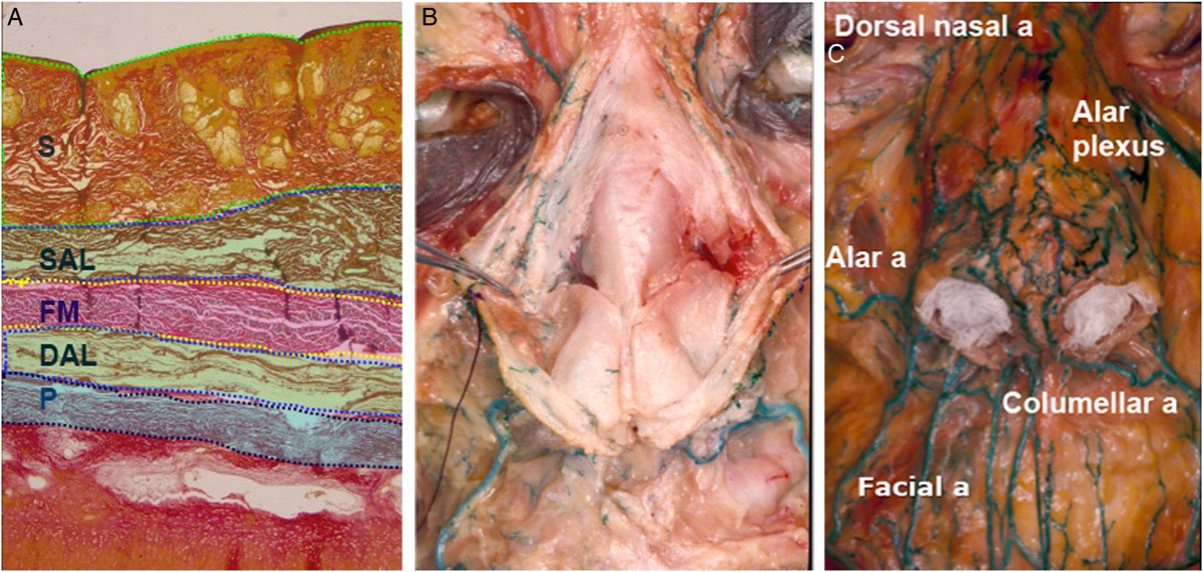
(A) Cross-section of soft tissue layers overlying the nasal osseocartilaginous framework. (B) The fibromuscular layer (FM) is a continuous sheet of muscle and aponeuroses that lies over the osseocartilaginous framework, sandwiched between the subcutaneous areolar layer (SAL) and the deep areolar layer. The FM is reflected in the midline showing that no major vessels lie beneath it. (C) The major vessels of the nose lie on the FM just under the SAL. These vessels comprise the paired alar and columellar arteries which are branches of the facial artery (derived from the external carotid system) and the paired dorsal nasal arteries which are branches of the ophthalmic artery (internal carotid system) and the plexus of anastomoses between them. DAL, deep areolar layer; FM, fibromuscular layer; P, perichondrium; S, skin; SAL, subcutaneous areolar layer.

As such, it can be concluded that the safest place to inject any fat or filler material into the nose is in the midline and as deep on the periosteum as possible. This is best achieved by using a sharp 30G needle inserted perpendicular to the skin and with the needle tip resting on the bone. With current technology, it is near impossible to inject fat particles out of anything less than a 26G needle which is why wider bore cannulae are chosen. However, it is difficult to direct a cannula whether inserted through the tip or the glabellar area down to the bony dorsum when its natural tendency is to enter and run in the planes of least resistance (ie, the two areolar layers with the fibromuscular layer sandwiched in between). Since the major blood vessels of the nose lie within this tissue sandwich it makes them vulnerable to perforation and embolization.

In the current paper,^5^ where 198 patients had no complications whatsoever (which is to be congratulated), it would be interesting to know what precautions the authors took to avoid these complications. Were local blocks with lidocaine and adrenaline used to create local vasoconstriction? Was the use of an 18G cannula in fact a safer choice as it is unlikely to penetrate any of the nasal vessels which are smaller in size? Can the authors attribute any benefits of the MAFT gun (such as the size of the particles) in helping to reduce the risk of such a complication? Or was it just due to careful technique and low injection pressure? What would the authors have done if a complication had in fact happened? These are unanswered questions but they should be addressed in any paper that discusses injections of fat or filler into the nose and periorbital regions in the light of Beleznay's review and other articles documenting blindness from such injections.^9-18^

Notwithstanding these omissions, the paper was an enjoyable and stimulating read.

## 

### Disclosures

The author declared no potential conflicts of interest with respect to the research, authorship, and publication of this article.

### Funding

The author received no financial support for the research, authorship, and publication of this article.

## References

[1] Wu WTL Chapter 8: Periorbital rejuvenation with injectable fillers. In: Cohen SR, Born TM, eds. Techniques in Aesthetic Plastic Surgery Series: Facial Rejuvenation with Fillers. New York, NY: Saunders/Elsevier; 2009:93-106.

[2] Liew S, Wu WT, Chan HH et al Consensus on Changing Trends, Attitudes, and Concepts of Asian Beauty. Aesthetic Plast Surg. 2015 Sep 25. DOI:10.1007/s00266-015-0562-0 [Epub ahead of print]. 10.1007/s00266-015-0562-0 PMC4819477 26408389

[3] Wu WT, Liew S, Chan HH et al Consensus on Current Injectable Treatment Strategies in the Asian Face. Aesthetic Plast Surg. 2016 Feb 18. DOI:10.1007/s00266-016-0608-y [Epub ahead of print]. 10.1007/s00266-020-01818-8 32844269

[4] Wu WTL. Aesthetic contouring of the upper third of the face with soft tissue fillers - a personal technique to improve volume deficits of the forehead, brow, upper lids, nose and temples. In: Tonnard P, Verpaele A, eds. *Centrofacial Rejuvenation*. Boca Raton, FL: CRC Press; 2016: (In press).

[5] Kao WP, Lin YN, Lin TY et al Microautologous Fat Transplantation for Primary Augmentation Rhinoplasty: Long-Term Monitoring of 198 Asian Patients. Aesthet Surg J*.* 2016;36 6:648-656. 26764261 10.1093/asj/sjv253 PMC5127412

[6] Coleman SR Structural fat grafting. Aesthet Surg J. 1998;18 5:386-388. 19328166 10.1016/S1090-820X(98)70098-6

[7] Beleznay K, Carruthers JD, Humphrey S, Jones D Avoiding and Treating Blindness From Fillers: A Review of the World Literature. Dermatol Surg. 2015;41 10:1097-1117. 26356847 10.1097/DSS.0000000000000486

[8] Wu WT The Oriental nose: an anatomical basis for surgery. Ann Acad Med Singapore. 1992;21 2:176-189. 1519881

[9] Park SW, Woo SJ, Park KH, Huh JW, Jung C, Kwon OK Iatrogenic retinal artery occlusion caused by cosmetic facial filler injections. Am J Ophthalmol. 2012;154 4:653-662.e1. 22835509 10.1016/j.ajo.2012.04.019

[10] Lazzeri S, Figus M, Nardi M, Lazzeri D, Agostini T, Zhang YX Iatrogenic retinal artery occlusion caused by cosmetic facial filler injections. Am J Ophthalmol. 2013;155 2:407-408. 23312444 10.1016/j.ajo.2012.10.012

[11] Carruthers JD, Fagien S, Rohrich RJ, Weinkle S, Carruthers A Blindness caused by cosmetic filler injection: a review of cause and therapy. Plast Reconstr Surg. 2014;134 6:1197-1201. 25415089 10.1097/PRS.0000000000000754

[12] He MS, Sheu MM, Huang ZL, Tsai CH, Tsai RK Sudden bilateral vision loss and brain infarction following cosmetic hyaluronic acid injection. JAMA Ophthalmol. 2013;131 9:1234-1235. 24030337 10.1001/jamaophthalmol.2013.1603

[13] Kim EG, Eom TK, Kang SJ Severe visual loss and cerebral infarction after injection of hyaluronic acid gel. J Craniofac Surg. 2014;25 2:684-686. 24621723 10.1097/SCS.0000000000000537

[14] Kim YJ, Kim SS, Song WK, Lee SY, Yoon JS Ocular ischemia with hypotony after injection of hyaluronic acid gel. Ophthal Plast Reconstr Surg. 2011;27 6:e152-e155. 10.1097/IOP.0b013e3182082f37 22082564

[15] Lazzeri D, Agostini T, Figus M, Nardi M, Pantaloni M, Lazzeri S Blindness following cosmetic injections of the face. Plast Reconstr Surg. 2012;129 4:995-1012. 22456369 10.1097/PRS.0b013e3182442363

[16] Liu OG, Chunming L, Juanjuan W, Xiaoyan X Central retinal artery occlusion and cerebral inrfaction following forehead injection with a corticosteroid suspension for vitiligo. Indian J Dermatol Venereol Leprol. 2014;80 2:177-179. 24685869 10.4103/0378-6323.129416

[17] Jiang X, Liu DL, Chen B Middle temporal vein: a fatal hazard in injection cosmetic surgery for temple augmentation. JAMA Facial Plast Surg. 2014;16 3:227-229. 24577714 10.1001/jamafacial.2013.2565

[18] Tansatit T, Apinuntrum P, Phetudom T An anatomical study of the middle temporal vein and the drainage vascular networks to assess the potential complications and the preventive maneuver during temporal augmentation using both anterograde and retrograde injections. Aesthetic Plast Surg. 2015;39 5:791-799. 26174139 10.1007/s00266-015-0529-1

